# Embedding FAIR Data Practices in Shared Core Resource Facilities

**DOI:** 10.7171/001c.167764

**Published:** 2026-09-01

**Authors:** Mousumi Ghosh, Ying Huang, Emily S. Boja, Jaime M. Guidry Auvil, Subhashini Jagu

**Affiliations:** 1 NCI Office of Data Sharing National Institutes of Health

**Keywords:** Shared Resources, Cancer Centers, NCI-designated Cancer Centers, Data Management and Sharing, FAIR data practices, core-generated datasets, data producers, metadata, data stewardship

## Abstract

Data fragmentation, lack of standardization, and incomplete clinical context limit the utility and reuse of datasets in biomedical research. Core facilities (shared resources) generate a large portion of research data and are well positioned to implement FAIR (Findable, Accessible, Interoperable, and Reusable) data practices to align with the NIH Data Management and Sharing Policy. This article highlights strategies for incorporating FAIR data practices into core facility workflows.

Core facilities generate diverse data types across multiple platforms. However, variability in data formats and metadata practices can pose challenges for interoperability and cross-study integration. A critical first step in addressing this challenge is to connect core-generated datasets derived from patient biospecimens with structured clinical metadata. This involves using standardized identifiers, integration with biobanks and electronic health records. Structured metadata capture during experimental workflows to ensure data provenance will enable meaningful downstream analysis.

Equally important is the adoption of standardized data formats and metadata frameworks across technologies. Adoption of community standards for various data types, along with standard metadata schemas, improves data discoverability and reuse. Robust institutional infrastructure (e.g. laboratory information management systems, integrated data platforms) and coordinated governance frameworks can help to implement these efforts. Partnerships with institutional stakeholders and targeted workforce training further support the consistent implementation of effective data stewardship practices.

These approaches position core facilities as central operational hubs for advancing FAIR data principles, enhancing reproducibility, and maximizing the long-term value of biomedical research data.

## Introduction

Shared instrumentation and core facilities are foundational components of the modern biomedical research enterprise as they centralize advanced technologies, specialized expertise, and high-value instrumentation. These facilities enable investigators across disciplines to access capabilities that would otherwise be beyond the reach of individual laboratories. As hubs within the institutional research ecosystem, core facilities foster collaboration and team science by connecting researchers, technologies, and data across laboratories and departments. They also play a critical educational role by introducing investigators and trainees to emerging technologies and providing training in experimental design, data generation, and analysis. By adopting standardized protocols and best practices, core facilities contribute significantly to research rigor, reproducibility, and transparency.^1–7^

The increasing scale and complexity of data generated by modern biomedical technologies further elevate the importance of shared core resource facilities in shaping institutional data management practices.^6^ Because cores generate a substantial portion of institutional research data across platforms, such as genomics, proteomics, and imaging, they are uniquely positioned to embed best practices for data stewardship directly into experimental workflows. Core facilities can ensure clear data provenance and readiness for downstream sharing and reuse by implementing standardized operating procedures, structured metadata capture, and consistent documentation of analytical parameters.

Importantly, shared resource facilities represent a strategic leverage point for advancing FAIR data practices across institutions. Operating as centralized environments that serve diverse research communities, cores can implement consistent workflows for metadata capture, data quality control, and documentation across multiple projects. Through their consultant role in experimental design and user training, core staff can introduce responsible data management practices at the earliest stages of research. By embedding FAIR-aligned data practices^8^ into routine service operations, core facilities can help operationalize emerging policy expectations — such as the National Institutes of Health (NIH) Data Management and Sharing Policy^9^ — while fostering a broader cultural shift toward more transparent, interoperable, and reusable research data.^10–14^ Three important steps in achieving this goal include: (1) connecting clinical information and patient data to biosample-derived data generated at core facilities, (2) encouraging the use of standardized formats for datatypes and minimum metadata, and (3) institutional support and infrastructure that enables these efforts.

## Connecting Shared Core Resource-Generated Data with Clinical Information

A critical first step in operationalizing FAIR data practices in shared resource facilities is ensuring that experimental data generated from patient-derived biospecimens are appropriately linked to relevant clinical information.^15^ Many technologies housed in core facilities — including genomics, proteomics, imaging, and other high-throughput platforms — analyze samples derived from tissues, blood, or other biospecimens obtained from patients during clinical care or clinical studies. While the resulting molecular or imaging datasets are often deposited in repositories or shared through publications, the absence of accompanying clinical context substantially limits their interpretability and downstream reuse. To enable meaningful secondary analysis and integration across studies, experimental data must be connected to patient-derived clinical metadata describing the individuals from whom the biospecimens were obtained.^16,17^

Recent workshops convened by the National Cancer Institute (NCI) Office of Data Sharing identified a core set of clinical features that are broadly required to support translational and clinical cancer research using human-derived data.^18^ These features span several major categories, including demographics, disease characteristics, molecular and diagnostic profiles, treatment exposures, and clinical outcomes (Table 1). Establishing systematic mechanisms for connecting experimental outputs generated in core facilities to structured clinical metadata is a key operational challenge. One practical approach may be to establish metadata pipelines that connect biospecimen records and clinical data sources to experimental datasets, using laboratory information management systems (LIMS).

**Table 1.** Minimum set of clinical metadata elements relevant to clinical research

**Clinical Data Category**	**Representative Data Elements**	**Relevance for Downstream Analysis**
**Demographics**	Age at diagnosis, sex, race/ethnicity	Enables population stratification, epidemiologic analysis, and identification of disparities in disease incidence and outcomes
**Disease Characteristics**	Cancer type, primary tumor site, histology/morphology, stage, grade, basis of the diagnosis (i.e., pathology, molecular characterization)	Provides essential clinical context for interpreting molecular or imaging data and defining disease subtypes
**Molecular and Diagnostic Features**	Tumor biomarkers, genomic alterations, molecular subtype, diagnostic assay results	Allows integration of clinical and molecular data and supports precision oncology research
**Treatment Information**	Treatment modality (surgery, radiation, systemic therapy), treatment regimens, dates of therapy, line of therapy	Enables evaluation of treatment response and association of molecular features with therapeutic outcomes
**Clinical Outcomes**	Response to therapy, disease progression, recurrence, survival status, progression-free survival	Critical for linking molecular findings to clinical endpoints and evaluating prognostic or predictive biomarkers
**Medical History and Comorbidities**	Prior cancer history, comorbid conditions, family history	Supports interpretation of disease risk factors and patient heterogeneity
**Social and Environmental Factors**	Socioeconomic indicators, lifestyle factors (diet, smoking, physical activity), environmental exposures	Important for understanding cancer risk, treatment access, and outcomes
**Healthcare Delivery Context**	Care setting (inpatient/outpatient), institution type, treatment facility characteristics	Enables implementation science and health systems research
**Longitudinal Clinical Data**	Follow-up visits, disease course over time, updated outcomes	Enables studies of disease progression, survivorship, and long-term treatment effects

## Standardizing Data Formats and Metadata to Enable Interoperability

**Figure 1.**
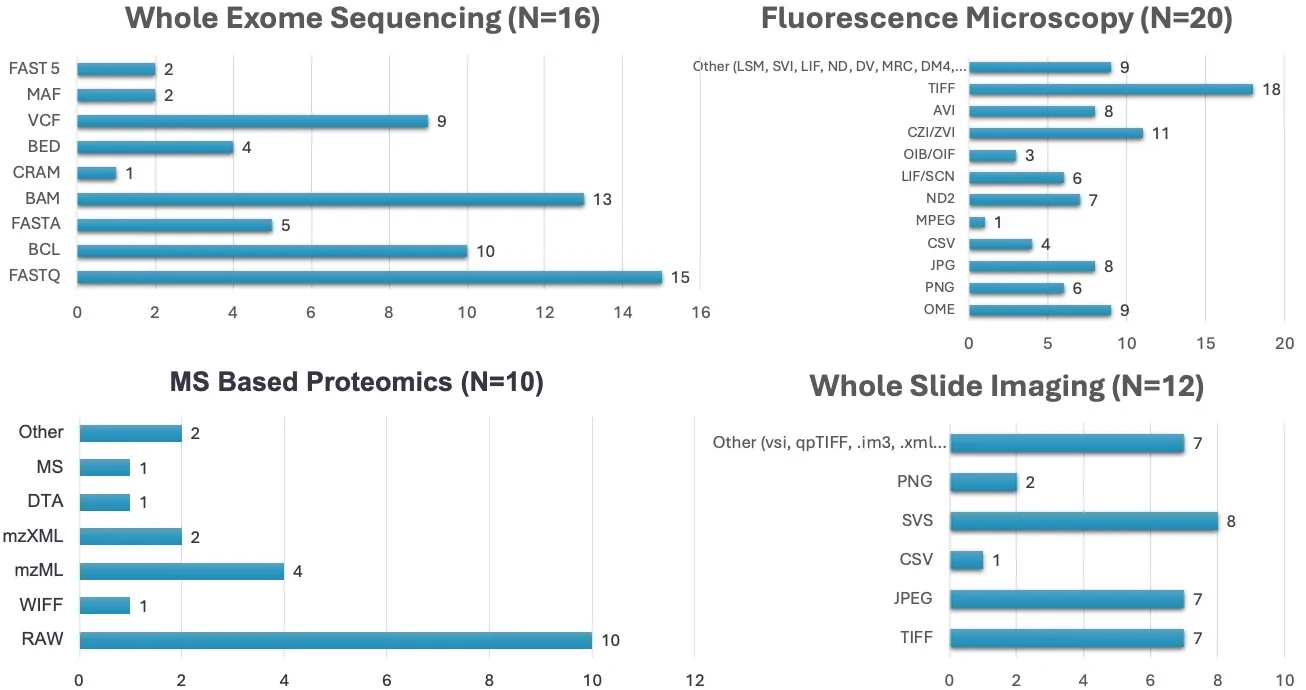
Data format heterogeneity across technologies in shared resource facilities across genomic, proteomic, metabolomic, microscopy and histology data.

In addition to connecting experimental data with clinical metadata, a critical requirement for making core-generated datasets FAIR is to adopt standardized data formats. A recent survey of shared instrumentation cores at cancer centers by the NCI Office of Data Sharing demonstrated that data produced by shared resource facilities, including proteomics, metabolomics, genomics, and imaging data, are typically generated using vendor-specific instruments and proprietary software (Figure 1). This data are then mainly shared in heterogeneous formats, making it difficult to integrate datasets across platforms, institutions, and studies. As a result, even when datasets are publicly shared, the lack of standardized formats can significantly limit their discoverability, interoperability, and reuse.

Equally important is the structured capture of metadata during the experimental workflow. Core facilities routinely collect instrument settings, assay conditions, and analytical parameters as part of standard operating procedures. Extending these workflows to include structured fields for sample preparation details, and study-level metadata ensures that datasets can be accurately contextualized and linked to laboratory data maintained elsewhere in institutional data systems such as electronic laboratory notebooks (ELNs). These practices also support provenance tracking, which is critical for reproducibility and downstream data integration.^19^

Adopting community-driven standards is essential for addressing these challenges. In several technology domains commonly supported by shared resource facilities, well-established standards have already been developed. For example, the Human Proteome Organization Proteomics Standards Initiative (HUPO-PSI) has introduced formats such as mzML,^20^ mzIdentML,^21^ and mzTab^22^ to standardize mass spectrometry data and other associated metadata.^23,24^ Similarly, the metabolomics community has promoted standardized reporting and data exchange frameworks through initiatives such as the Metabolomics Standards Initiative (MSI)^25,26^ and repositories including MetaboLights. In imaging, the Open Microscopy Environment (OME) has developed interoperable file formats such as OME-TIFF and OME-NGFF,^27^ along with metadata guidelines such as REMBI (Recommended Metadata for Biological Images)^28^to facilitate consistent data annotation and sharing.

Despite the availability of these frameworks, adopting them across laboratories and instrument vendors remains uneven.

Shared resource facilities can play a pivotal role in advancing data interoperability by embedding standards-compliant workflows into routine service pipelines. For example, core facilities can implement automated export pipelines that convert proprietary instrument outputs into community-supported formats, incorporate standardized metadata templates into sample submission workflows, and provide guidance to investigators on selecting appropriate repositories and data standards for their datasets. By integrating these practices directly into the data generation process, core facilities can ensure that datasets produced across institutional research programs are structured in ways that enable seamless discovery, integration, and reuse.

## Building an Institutional Infrastructure to Support FAIR Data Generation in Shared Resource Facilities

Operationalizing FAIR data practices within shared resource facilities requires more than just adopting standards and capturing appropriate metadata; it also depends on the availability of institutional infrastructure that supports consistent data management across the research lifecycle. Because core facilities generate large volumes of highly structured experimental data across multiple technologies, they are uniquely positioned to serve as integration points between laboratory workflows and institutional data ecosystems. Establishing the appropriate technical and organizational infrastructure (such as LIMS systems) that are integrated with ELNs and biobanks can enable these facilities to efficiently capture, manage, and disseminate research data in ways that align with FAIR principles and funder expectations.^17,29,30^

Furthermore, partnerships with institutional libraries, research informatics groups, and information technology departments^31^ can support the development of data repositories, metadata catalogs, and data discovery tools that improve the findability and accessibility of core-generated datasets. Institutional data commons^32^ initiatives and federated data platforms^33^ also provide mechanisms for harmonizing datasets across multiple research programs, which enable investigators to identify and reuse data generated across different laboratories and technologies.

Finally, core facility staff routinely provide technical training to investigators and trainees on experimental methods and instrument operation; these interactions also represent valuable opportunities to introduce best practices for data management, metadata documentation, and data sharing. By integrating data stewardship into routine research workflows and training activities, shared resource facilities can help cultivate a research culture in which FAIR data practices are viewed as a standard component of high-quality scientific work.

Together, these institutional infrastructure elements — data management systems, coordinated governance structures, and workforce training — create the foundation needed for shared resource facilities to generate datasets that are well-documented, interoperable, and ready for responsible sharing. Investments in these capabilities will be essential for enabling core facilities to fulfill their role as operational engines for FAIR data generation.

## Conclusion

In conclusion, shared resource facilities — such as genomics, proteomics, imaging, and other biophysical cores — play a vital role in operationalizing federal data sharing policy. These facilities generate large-scale datasets whose downstream value depends on consistent linkage to a well-defined clinical context and other metadata. Furthermore, standardization of data across core facilities is critical for the data to be used together. Aligning shared resource practices with NIH data sharing expectations can improve compliance, enhance interoperability, and increase the long-term impact of publicly funded cancer research.
